# Movement through Active Personalised engagement (MAP) — a self-management programme designed to promote physical activity in people with multimorbidity: study protocol for a randomised controlled trial

**DOI:** 10.1186/s13063-018-2939-2

**Published:** 2018-10-20

**Authors:** Helen Dallosso, Tom Yates, Hamidreza Mani, Laura J. Gray, Nafeesa Dhalwani, Emma Baldry, Clare Gillies, Sue Cradock, Mark Batt, Melanie J. Davies, Kamlesh Khunti

**Affiliations:** 1 Leicester Diabetes Centre, University Hospitals of Leicester, Leicester General Hospital, Leicester, LE5 4PR UK; 2 Diabetes Research Centre, College of Medicine, Biological Sciences and Psychology, University of Leicester, Leicester, LE5 4PW UK; 3 NIHR Leicester Biomedical Research Centre, University of Leicester, Leicester, UK; 4 Department of Health Sciences, College of Life Sciences, University of Leicester, Leicester, LE1 7RH UK; 5 Centre for Sports Medicine, Nottingham University Hospitals NHS Trust, Nottingham, NG7 2UH UK; 6 NIHR Collaboration for Leadership in Applied Health Research and Care - East Midlands, University of Leicester, Leicester, UK

**Keywords:** Randomised controlled trial, Primary care, Multimorbidity, Physical activity, Self-management, Patient education

## Abstract

**Background:**

Multimorbidity, defined as two or more concurrent chronic diseases within the same individual, is becoming the clinical norm within primary care. Given the burden of multimorbidity on individuals, carers and health care systems, there is a need for effective self-management programmes. Promoting active participation within their clinical care and following a healthy lifestyle will help empower patients and target lifestyle factors that are exacerbating their conditions. The aim of this study is to establish whether a tailored, structured self-management programme can improve levels of physical activity at 12 months, in people with multimorbidity.

**Methods/design:**

This study is a single-centre randomised controlled trial, with follow-up at 6 and 12 months. The primary outcome is change in objectively assessed average daily physical activity at 12 months. Secondary outcomes include medication adherence, lifestyle behaviours, quality of life, chronic disease self-efficacy and self-efficacy for exercise. Anthropometric and clinical measurements include blood pressure, muscle strength, lipid profile, kidney function and glycated haemoglobin (HbA1c). Participants are recruited from primary care. Those between 40 and 85 years of age with multimorbidity, with a good understanding of written and verbal English, who are able to give informed consent, have access to a mobile phone for use in study activities and are able to walk independently will be invited to participate. Multimorbidity is defined as two or more of the chronic conditions listed in the Quality and Outcomes Framework. A total of 338 participants will be randomly assigned, with stratification for gender and ethnicity, to either the control group, receiving usual care, or the intervention group, who are invited to the Movement through Active Personalised engagement programme. This involves attending four group-based self-management sessions aimed at increasing physical activity, mastering emotions, managing treatments and using effective communication. The sessions are delivered by trained facilitators, and regular text messages during the study period provide ongoing support. Changes in primary and secondary outcomes will be assessed, and an economic evaluation of the intervention undertaken.

**Discussion:**

This study will provide new evidence on whether physical activity can be promoted alongside other self-management strategies in a multimorbid population and whether this leads to improvements in clinical, biomedical, psychological and quality of life outcomes.

**Trial registration:**

ISRCTN, ISRCTN 42791781. Registered on 14 March 2017.

**Electronic supplementary material:**

The online version of this article (10.1186/s13063-018-2939-2) contains supplementary material, which is available to authorized users.

## Background

Multimorbidity, defined by the World Health Organization as two or more concurrent chronic health conditions within the same individual [1], is becoming the clinical norm within the ageing population in primary care [2]. In developed countries the prevalence of multimorbidity in the general population is estimated to be between 25 to 55% at 60 years of age and as high as 80% in those older than 75 years [3, 4]. Patients with multimorbidity are more likely to die prematurely, be admitted to hospital and have longer hospital stays than those with single conditions [5, 6]. Multimorbidity leads to a decline in functional status and quality of life, and patients are more likely to experience depression and to be receiving multiple drugs with consequent difficulties with adherence [7, 8]. Multimorbidity increases health care expenditure compared to that for patients with a single chronic disease and requires bespoke systems of management [9]. However, despite this new clinical reality, routine health care systems are still largely based around single disease management pathways and guidelines, which can result in overtreatment or sub-optimal treatment and fragmented care. These problems are exacerbated in those who are elderly [10], from areas of high social deprivation [11] or from black and minority ethnic backgrounds [12].

An effective self-management and healthy lifestyle programme helps in empowering patients and facilitating active participation within their clinical care and targeting lifestyle factors that are exacerbating their conditions. Self-management programmes are an integral part of many disease management pathways and have been recommended in the UK by the National Institute for Health and Care Excellence (NICE) [13–17]. However, self-management and lifestyle interventions have not been specifically tailored or implemented for use in those with multimorbidity and are not mentioned in the NICE Guidelines for the Management of Multimorbid Conditions [9].

Physical inactivity is associated with an increased risk of all-cause mortality, cardiometabolic disease, musculoskeletal problems, declining cognitive function, poor psychological health and reduced quality of life [18]. Therefore, given these wide-ranging links across the chronic disease spectrum, it is unsurprising that physical activity has also been associated with multimorbidity [19–21]. It has been shown that an increase in ambulatory activity of 2000 steps/day is associated with an 8% reduction in the relative risk of cardiovascular mortality or morbidity in those with prediabetes and existing cardiovascular disease [22]. Others have shown that physical activity-based interventions have a similar level of efficacy compared to established pharmaceutical therapies in the secondary prevention of cardiovascular disease, whilst also effectively reducing symptoms of depression in those with chronic disease, improving measures of functional ability and improving cognitive function [23–25]. Improvements in physical functioning following physical activity interventions have been observed in multimorbidity [26]. However, few trials have looked at the effectiveness of interventions designed to improve lifestyle outcomes in patients with multimorbidity in primary care [27], and the evidence related to changes in lifestyle behaviours such as diet and physical activity is conflicting [28–30]. This observational and interventional evidence, across multiple outcomes and conditions, suggests that physical activity can be conceptualised as a composite marker of overall health status. A positive change in physical activity would therefore ensure an intervention targets many of the clinical factors associated with multimorbidity. The present study aims to develop and evaluate a self-management programme for patients with multimorbidity that focusses on lifestyle factors such as physical activity, medication adherence and self-management and can be translated into primary care.

## Methods/design

### Aims and objectives

The aims of this trial are to:

1. Test the effectiveness of a structured self-management programme on increasing the levels of physical activity using accelerometry at 12 months in people with multimorbidity
2. Assess the effectiveness of the programme on improving clinical measures including blood pressure, lipid profile, body weight and grip strength
3. Assess the effectiveness of the programme on improving self-management outcomes including medication adherence, lifestyle behaviours, clinical outcomes, quality of life and self-efficacy
4. Carry out an economic evaluation of the intervention. This will assess implementation and running costs, as well as short-term and long-term cost-effectiveness

### Study design

**Fig. 1:**
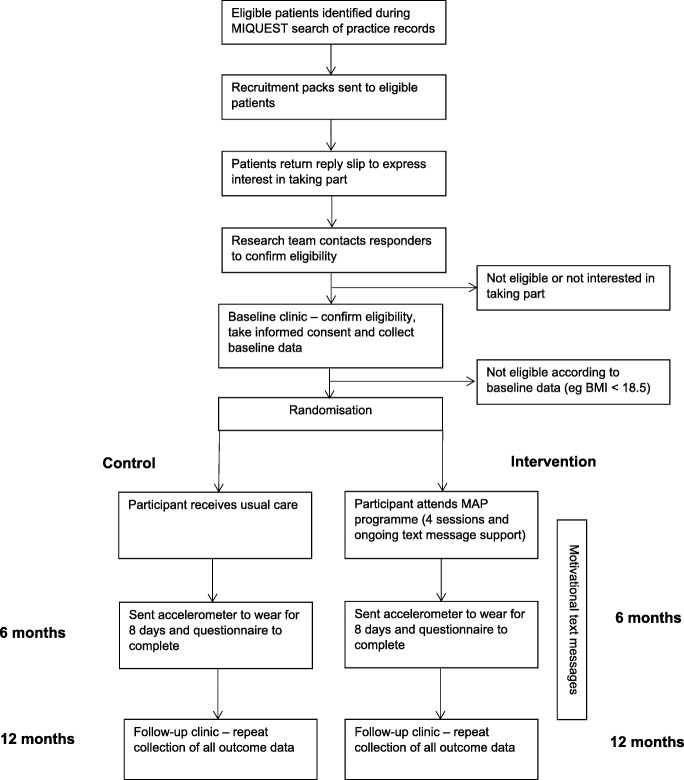
Schematic representation of study

The study is a single-site, two-arm, parallel, 12-month randomised controlled trial (RCT) testing the effectiveness of a tailored structured self-management programme. Participants are recruited from primary care in Leicestershire, UK. For the purposes of the trial, multimorbidity is defined using the most common definition, i.e. two or more long-term conditions [11, 31]. Figure 1 describes the study flow and participant progression through the study. The study is sponsored by the University of Leicester, and ethical approval was granted by West Midlands – South Birmingham Research Ethics Committee and the Health Research Authority. The study was prospectively registered (ISRCTN 42791781), and the completed Standard Protocol Items: Recommendations for Interventional Trials (SPIRIT) checklist is available as an additional document (Additional file 1).

### Recruitment and informed consent

Potential participants with multimorbidity (see the next section for the definition) are identified by a bespoke electronic Morbidity Information Query and Export Syntax (MIQUEST) search [32] of the practice’s electronic health records, and the list of potential participants is reviewed by an appropriate member of the practice staff. Potential participants are then sent a recruitment pack containing an invitation letter, a brief information leaflet detailing the study and a reply slip. Reply slips from those interested in taking part are returned to the study team. A member of the study team then contacts the patient by telephone to screen them and arrange a baseline visit to formally confirm eligibility, undertake informed consent and collect the baseline data. Following the telephone screening, the patient is sent the full Participant Information Leaflet (PIL) and a letter confirming the details of the baseline clinic. These documents are sent at least 5 days before the appointment to ensure that patients have a minimum of 24 h to read the PIL and ask questions of the study team prior to giving consent. Recruitment clinics are staffed by trained research nurses and health care assistants employed by the University Hospitals of Leicester National Health Service (NHS) Trust, and the clinics are held in local community venues. After confirming the patient’s eligibility, written informed consent is taken by a member of the team trained in the procedure.

### Inclusion and exclusion criteria

For the purpose of the study, multimorbidity is defined as having two or more of the chronic conditions included in the Quality and Outcomes Framework (QOF) [33] with the exception of those classified under palliative care or within the mental health and neurology category, other than depression (Table 1). These conditions are excluded given their complexity and specialist needs, which are likely to be beyond general group-based self-management interventions. ’Primary prevention of cardiovascular disease’ is not included as a chronic condition, as patients under this category would already have a diagnosis of hypertension and/or type 2 diabetes. Obesity is not considered as a separate condition, since it is ubiquitous in many chronic diseases [34]. Patients undergoing treatment for cancer are excluded, but if in remission they can take part if they have two other chronic conditions. Patients with both asthma and chronic obstructive pulmonary disease are included only if they have another morbidity. There are no limitations concerning disease duration, medication use and/or initiation of new treatment unless there are specific recommendations for bed rest and minimising physical activity for a specific condition.

**Table 1:** Conditions included in the Quality and Outcomes Framework (QOF) [33]

Cardiovascular	Secondary prevention of coronary heart disease
	Cardiovascular disease, primary prevention*
	Peripheral arterial disease
	Atrial fibrillation disease
	Heart failure
	Hypertension
	Stroke/transient ischaemic attack
Respiratory	Asthma**
	Chronic obstructive pulmonary disease**
Lifestyle	Obesity*
High dependency and long-term conditions	Cancer***
	Chronic kidney disease
	Diabetes mellitus
	Hypothyroidism
	Palliative care****
Mental health and neurology	Depression
	Dementia****
	Learning disability****
	Mental health****
	Epilepsy****
Musculoskeletal	Osteoporosis
	Rheumatoid arthritis

*Patients with these conditions require at least two other conditions in order to be included

**Patients with both asthma and chronic obstructive pulmonary disease require at least one other condition to be included

***Patients undergoing cancer treatment are excluded. If treatment is finished they can participate, but two other conditions are required to be included

****Patients with these conditions are excluded

In addition, patients must be aged between 40 to 85 years of age inclusive, have a good understanding of written and verbal English, be able to give informed consent, have access to a mobile phone for use in study activities, be willing and able to attend the recruitment clinics and self-management sessions and be able to walk independently. Patients who have limited understanding of written and verbal English, are pregnant or are currently participating or have participated in another interventional trial in the previous 12 weeks are excluded. Patients with frailty (defined as one or more of the following: living in care homes or institutions; needing daily support for activities such as washing, cooking and household tasks; having had unintentional significant weight loss in the last 3 to 6 months; having a body mass index of less than 18.5 kg/m^2^) are also excluded from the study.

### Randomisation and blinding

Study identification numbers are assigned sequentially, and participants are individually randomised (1:1) stratified by gender (men; women) and ethnicity (White European; other) using a variable block size after their baseline assessment. In order to prevent contamination, an exception is made for individuals from the same household who are allocated to the same group. The randomisation schedule was developed by an independent statistician, and allocation of randomisation is carried out by a researcher independent of the team. Randomisation dictates whether the participant receives standard care or is invited to attend the Movement through Active Personalised engagement (MAP) programme.

After randomisation both groups are sent a letter informing them of the outcome of the randomisation; in addition, intervention participants are contacted by telephone to discuss the dates available for them to attend the intervention. They are then sent a letter confirming the venue, dates and times of the programme. Since the intervention is a group self-management programme, participants and the central study team cannot be blinded to the randomisation. The research nurses who run the follow-up clinics are not informed of the randomisation of the participants attending, and staff analysing accelerometer data to derive the primary outcome are blinded.

### Treatment regimens

There is no interference with the ongoing medical treatment, clinical care or planned follow-up appointments of the participants in primary or secondary care.

#### Control group

Participants continue with their routine care management in line with their clinical care team’s current recommendations.

#### Intervention group

Those randomised to the intervention group are invited to attend the MAP programme. The programme comprises four facilitated group sessions delivered in local community settings. The sessions are run approximately 2 weeks apart and last about 1.5 h. The first session takes place within approximately 1 month of consent.

The MAP programme was developed using an iterative process, including a literature review to identify effective strategies within this population, and co-production with patient and public involvement (PPI) and other stakeholder groups (including clinicians and nurses who work in primary care). The literature review guided a content focus on the key generic challenges for this patient group: mastering emotions, managing treatments and communicating with a range of carers (family and health care professionals). The group discussions informed the content and format of the programme to include both a focus on increasing physical activity as well as the generic self-management challenges. The design of the programme was theoretically informed by social learning theory [35] and focussed carefully on the role of the facilitator to support participants to explore their own beliefs, understanding and concerns related to living with two or more long-term conditions. The design of the programme was underpinned by a fundamental belief that individuals know what is best for their health, yet may be more informed in their decision-making by taking part in an open and honest discussion that focusses on increased knowledge and insight through shared experiences [36].

A draft programme was tested with patient volunteers using an iterative process of testing, feedback and modification until the programme was completed and ready to be utilised in the RCT. Given the diverse patient group, the primary focus of the MAP programme is on factors related to development of health-related self-management behaviours, rather than being disease-specific. Each of the four sessions focusses discussion on moving more as well as on a session-specific topic related to the generic self-management challenges: mastering emotions, managing treatments and effective communication. Increasing physical activity is encouraged by using pedometers and exercise resistance bands, which are given to intervention participants. All sessions include a section on physical activity, with the later sessions focussing on reviewing goals and progress. The main format is group discussion, but the programme is person-centred, with self-monitoring and individual goal setting a key part. Additional file 2 summarises the structure and content of the four sessions.

The sessions are delivered by a ‘non-disease specific expert’ facilitator who has received training on the content and ‘person-centred’ delivery style of the programme [37]. Adopting a person-centred delivery style of the sessions focusses the need for facilitators to use behaviours that support participant engagement and reflection by all participants. Facilitators are provided with a specific training curriculum to support the delivery of education sessions. They are encouraged to use personal reflection and peer review to maintain the quality of the delivery and are supported with mentorship from trainers attached to the research team. Delivery of a number of sessions will be assessed by a trained observer in order to provide a measure of intervention fidelity. A structured observation tool will be used to assess facilitator delivery of prescribed behaviours and behaviour change techniques [38], and the tool includes an assessment of ‘talk-time’ as a measure of quality of delivery [39].

Follow-up support to motivate and promote positive health behaviour changes [40, 41] and improved medication adherence [42] have been shown to be effective when provided as text messaging. In order to support long-term behaviour change, regular reminder and motivational text messages are sent using an established independent provider. The commencement, frequency and duration of the messages are based on the design protocols developed for other self-management programmes [43–45] as well as consultation with stakeholders. Message delivery is automated and unidirectional and incurs no cost to participants. Participants who wish to stop receiving the messages can text ‘STOP’ in a response text. Additional file 3 summarises the frequency and content of the messages.

### Study management

Clinical visits are managed by trained research staff, predominantly research nurses and health care assistants from the University Hospitals of Leicester NHS Trust. All research staff have been trained in and follow standard operating procedures (SOPs) when collecting the data. Written informed consent is obtained by a trained research nurse before any trial activities take place. Data are collected at three time points: baseline, 6 months (postal) and 12 months (Fig. 1 and Table 2). In addition to the primary and secondary outcome data, demographic data and medical history data (details of relevant history of disease, medications, relevant surgical interventions) are recorded.

**Table 2:** Summary of outcome assessment schedule

Outcome measures	Method	Baseline	6 months	12 months
Physical activity	Accelerometer worn for 8 continuous days and data downloaded	√	√	√
	Recent Physical Activity Questionnaire [49]	√	√	√
Self-efficacy	Chronic Disease Self-Efficacy Scale [56]	√	√	√
	Self-Efficacy for Exercise scale [57]	√	√	√
Quality of life	EuroQoL EQ-5D-5L scale. Health-Related Quality of Life Instrument [53]	√		√
	Hospital Anxiety and Depression Scale [54]	√		√
Medication adherence	Adherence Starts with Knowledge 12 questionnaire (ASK-12) [52]	√		√
Lifestyle	Diet, smoking status, sleeping behaviour questions	√		√
Demographic information	Ethnicity, work status, marital status questions	√		√ (except ethnicity)
Biochemical outcomes	Non-fasting blood sample to measure lipid profile, HbA1c, kidney function	√		√
Anthropometric measures	Height, weight, body mass index, waist and hip circumferences	√		√
Clinical measures	Blood pressure, pulse rate and grip strength	√		√
Medical history and medication	Self-reported	√		√
Use of health care services	Self-reported			√

### Primary outcome

The primary outcome measure is change in objectively measured physical activity from baseline to 12 months using the GENEActiv wrist-worn triaxial accelerometer (GENEActiv model 1.1, ActivInsights Ltd., Cambridgeshire, UK) with a dynamic range of +/− 8 *g*, where *g* is equal to the Earth’s gravitational pull. Participants are asked to wear the GENEActiv accelerometer on their non-dominant wrist for 8 consecutive days (24 h), wearing the monitor from the date of the assessment visit or from a specified date when sent in the 6 months postal follow-up. The accelerometer is initialised to collect data at 100 Hz. An appropriately trained individual instructs the participant on correct placement of the monitor. Participants are asked to complete a log whilst wearing the monitor to provide their waking hours and wear time information. Participants are given a stamped addressed envelope in which to return the monitor and log sheet once completed. Accelerometer data will be calibrated and analysed according to best practice procedures through the Lifestyle Theme of the National Institute for Health Research (NIHR) Leicester Biomedical Research Centre. In brief, data will be processed and calibrated using a bespoke open source package in R (GGIR, http://cran.r-project.org) [46, 47] according to criteria previously described [48]. Data will be included if participants have one or more valid days of data, with a valid day defined as at least 16 h of wear time. The primary outcome is defined a priori as average movement intensity as quantified by the Euclidean norm minus 1 *g* (ENMO) method. In addition, time asleep, sleep quality and time in sedentary behaviour; light-intensity physical activity; and moderate to vigorous physical activity will also be derived using validated algorithms and thresholds.

### Secondary outcomes

The following secondary outcomes are collected at baseline and at 12 months, and a number are also collected at 6 months by postal questionnaire (Table 2).

#### Clinical measures

Blood pressure and resting pulse rate are measured after the participant has been sitting for 5 min using the Omron HEM-907 Digital Upper Arm Cuff Blood Pressure Monitor (Omron Corporation, Kyoto, Japan). Three measurements are made, and the first measurement is excluded when calculating the mean. Grip strength is measured using a Baseline Hydraulic Hand Dynamometer (Fabrication Enterprises Inc., White Plains, NY, USA) according to the standardised procedure. Three measurements are made on both hands, and the highest value is used in the analysis.

#### Blood tests

Venous blood samples are taken and sent for analysis of full lipid profile, kidney function (sodium, potassium, urea, creatinine and estimated glomerular filtration rate (eGFR) and glycated haemoglobin (HbA1c) in accredited laboratories at University Hospitals of Leicester NHS Trust. The samples are analysed in accordance with the laboratory’s SOPs and destroyed after analysis. All results are reviewed by the study clinician. Where results are abnormal and clinically significant, historical results from the previous 6 months are reviewed. If there have been substantial changes in any of the levels, a letter is sent to the participant’s general practitioner.

#### Anthropometric measures

Body weight (in kilograms) is measured using the bioelectrical impedance Tanita Scales BC-418-MA (Tanita Corporation, Tokyo, Japan) and height (in metres) using a portable stadiometer (Holtain, Crymych,UK). Weight and height are used to calculate body mass index (weight in kilograms/height in metres squared). Waist circumference (centimetres) is measured at approximately 1 cm above the iliac crest and hip circumference at the widest area around the gluteus maximus.

#### Questionnaire measures

##### Recent Physical Activity Questionnaire (RPAQ)

The RPAQ is designed to explore day-to-day physical activity levels in the previous 4 weeks. The questionnaire is divided into three sections: (1) physical activity patterns in and around the house, (2) travel to work and work activities and (3) recreational activities. The RPAQ has reasonable validity for measuring total physical activity levels [49, 50].

##### Adherence Starts with Knowledge 12 (ASK-12) questionnaire

The ASK-12 questionnaire is used to assess medication adherence. It is a validated tool containing 12 questions and was adapted from the validated ASK-20 questionnaire [51, 52]. Twelve patient-specific barriers are scored from 1 to 5 using a Likert scale. Sub-scales providing measures of inconvenience/forgetfulness, treatment beliefs and behaviour can be calculated as well as a total score (ranging from 12 to 60).

##### EuroQoL five-dimensional, five-level version (EQ-5D-5L)

The EQ-5D assesses health-related quality of life and provides useful data for health economic analyses. The EQ-5D-5L is a validated measure of health status and has been validated specifically in chronic conditions, such as cardiovascular disease. The EQ-5D-5L has five quality of life dimensions (mobility, self-care, usual activities, pain/discomfort and anxiety/depression) which are all coded between 1 and 5 [53].

##### Hospital Anxiety and Depression Scale (HADS)

The HADS is a validated 14-item questionnaire measuring the severity of symptoms of anxiety and depression [54]. The anxiety and depression scores are each calculated from 7 questions which are scored from 0 to 3 on a Likert scale. Upon completion, selected scores are totalled and reported for anxiety and depression individually.

##### Chronic Disease Self-Efficacy Scale (CDSES)

The validated 33-item CDSES [55, 56] consists of three key concepts: self-efficacy to perform self-management behaviours, self-efficacy to manage disease in general and self-efficacy to achieve outcomes. In addition, the concepts are broken down into a number of sub-scales. All items are scored from 1 (not at all confident) to 10 (totally confident) using a Likert scale.

##### Self-Efficacy for Exercise (SEE) Scale

The SEE scale is a validated nine-item instrument that focusses on self-efficacy expectations related to the ability to continue exercising in the face of barriers to exercise [57]. The barriers are specifically related to weather, boredom, pain, exercising alone, lack of enjoyment, busyness, tiredness, stress and depression.

##### Lifestyle measures (diet, smoking status and alcohol consumption)

Dietary behaviour is captured using two short questionnaires based on dietary questionnaires developed for the European Prospective Investigation into Cancer and Nutrition (EPIC) study [58] and the international Nateglinide And Valsartan in Impaired Glucose Tolerance Outcomes Research (NAVIGATOR) study [59].

##### Use of health care services

These questions record the number of times in the past 12 months the participant has seen a general practitioner, practice nurse or other health care professional as well as details of prescribed and purchased medications and number of off-sick days.

### Economic evaluation

During the period of the trial the cost of running the MAP programme will be compiled from the unit costs mobilised in order to implement the service; this will include staff costs, room hire, costs of materials and resources utilised and participant travel costs. Data on health care utilisation will also be collected and will include both general practitioner and emergency department visits, hospitalisations and medication use. Research costs, such as those for clinic visits and blood tests, will be considered separately.

If the RCT finds no significant effect on clinical outcomes, a cost-consequence analysis will be undertaken. If a significant impact is found, cost-effectiveness analyses will be carried out for the outcomes that show a significant difference between the two trial arms. If the intervention shows a significant improvement in quality of life, then a cost-utility analysis will also be performed using preference-based health utility scores generated from the EQ-5D-5L questionnaire. Both a short-term cost-effectiveness analysis covering the period of the trial (12 months) and a Markov model using external data and projecting longer-term cost-effectiveness will be considered.

### Sample size

The primary outcome is change from baseline to 12 months in average daily physical activity as quantified by ENMO and measured in milligravitional units (m*g*). In order to detect a minimum clinically significant difference of 2.1 m*g*, which is equivalent to an overall increase in physical activity volume of approximately 10 metabolic equivalent hours per week and is consistent with minimum recommendations for health, and assuming a standard deviation of 5.3 m*g* [60], a power of 80% and a significance level of 5%, the sample size requires 202 participants. To allow for 20% loss to follow-up and 20% non-compliance of accelerometer/intervention attendance, we will need to recruit 338 participants (169 in each arm).

### Statistical analysis

A Consolidated Standards of Reporting Trials (CONSORT) diagram will summarise the flow of participants through the study. Descriptive characteristics at baseline will be summarised by arm. Numbers (with percentages) for binary and categorical variables, and means (and standard deviations) or medians (with lower and upper quartiles) as appropriate for continuous variables will be presented. Preliminary graphical and tabular presentations of the data will be inspected for the correct statistical modelling assumptions.

The primary analyses will use a complete case population (i.e. participants with a complete log and valid outcome measurements). For the primary outcome (change in average daily physical activity), the intervention group will be compared to the control using a linear regression model with a binary indicator for randomisation group as the explanatory variable; terms for the stratification factors (ethnicity and gender) as confounders; and adjustment for the change from baseline in accelerometer wear time and baseline average daily physical activity. Sensitivity analyses will include a per-protocol analysis and an intention-to-treat analysis where missing data will be imputed using multiple imputation or another suitable method. Sensitivity analysis will also test whether outcomes are robust if physical activity data are restricted to those with at least 3 days of valid data. Interaction effects will be fitted between intervention arm and gender (male vs. female), and ethnicity (White European vs. other). If the interaction term is statistically significant (at the 10% level), then stratified analyses will be performed for that factor using the same model as the primary analyses. Secondary outcomes will be analysed using similar methods as in the main analysis, with an appropriate model selected dependent on the distribution of the outcome. A detailed statistical analysis plan will be written and approved before the database is locked.

### Data management and monitoring

Data are entered on a validated electronic password protected database on a University of Leicester server, with only the participant identification number included. Hard copies of the data will be stored in locked filing cabinets and will be destroyed 10 years after the end of the study. The study is being conducted in accordance with the Research Governance Framework for Health and Social Care [61], International Conference on Harmonisation Good Clinical Practice (ICH GCP) guidelines and the Data Protection Act. As this is a minimal risk study, a Data Safety Monitoring Committee has not been convened. All staff working on the study have completed the required GCP training and follow the sponsor’s SOPs throughout the study. Serious adverse events (SAEs) are monitored and reported in line with requirements. An internal group meets every month to review recruitment rate, drop out, issues concerning delivery of the intervention and SAEs. A quarterly report on progress is submitted to the funder.

## Discussion

As the population of older people grows in developed nations, multimorbidity is becoming increasingly important in the health care landscape. Much of the management of multimorbidity is undertaken in primary care, and an effective group intervention would improve management and outcomes in this group of patients.

Physical activity is recommended as one of the main lifestyle changes in the management and prevention of multiple chronic disease [62], and its many beneficial effects have been demonstrated. Higher levels of physical activity are associated with lower rates of all-cause mortality and various morbidities [18]. Physical activity is also associated with quality of life, by increasing an individual’s strength, ability to perform daily chores and participate in social interactions, mobility and cognitive performance [34]. Cross-sectional evidence on its association with multimorbidity is inconclusive [63], and there is a need for longitudinal and interventional studies. To our knowledge, the MAP study will be the first interventional study to establish the effectiveness of a tailored programme to promote physical activity and engagement in self-management, through health-related and lifestyle behaviours, for people with multimorbidity. Participants are being identified in primary care, thus increasing the generalisability of the results, and the programme is being delivered in a ‘real world setting’ by trained ‘non-expert’ facilitators in a community setting.

Development of the programme included a large element of PPI work, with input provided by patients as well as health care professionals based in primary care. A training and mentoring programme has been developed, and the programme’s focus on aspects of intervention fidelity will highlight the ability of facilitators to adopt the ‘person-centred’ style and to envision what participants’ possible thoughts are as they leave the group sessions. The MAP self-management programme with its follow-on text messaging support system, if effective, is a model of care that can be scaled up and implemented in routine primary care.

### Trial status

Recruitment started on 14 June 2017 and is ongoing.

### Protocol version

The current version is Version 5; 2/2/2018. Three substantial amendments to the protocol have been approved. Amendment 1 (before recruitment started) involved a change in the procedure of randomisation (from an online software system to manual allocation using a randomisation schedule). Amendment 2 (before recruitment started) involved a change in choice of medication adherence scale. Amendment 3 (whilst recruitment was ongoing) involved adding an allowance for intervention non-adherence to the power calculation with a resultant increase in sample size.

## Additional files

Additional file 1: SPIRIT checklist. (DOC 120 kb)

Additional file 2: Structure and content of the four sessions. (PDF 1237 kb)

Additional file 3: MAP follow-on support pathway. (PDF 590 kb)

## Abbreviations

ASK-12: Adherence Starts with Knowledge 12 questionnaire
CDSES: Chronic Disease Self-Efficacy Scale
eGFR: Estimated glomerular filtration rate
ENMO: Euclidean norm minus 1 *g*
HADS: Hospital Anxiety and Depression Scale
HbA1c: Glycated haemoglobin
MAP: Movement through Active Personalised engagement
NICE: National Institute for Health and Care Excellence
PIL: Participant Information Leaflet
PPI: Patient and public involvement
QOF: Quality and Outcomes Framework
RCT: Randomised controlled trial
RPAQ: Recent Physical Activity Questionnaire
SAE: Serious adverse event
SEE: Self-Efficacy for Exercise (scale)
SOP: Standard operating procedure
