# Supplementary material for: Movement through Active Personalised engagement (MAP) — a self-management programme designed to promote physical activity in people with multimorbidity: study protocol for a randomised controlled trial
Source: Trials. 2018 Oct 20;19:576. doi: 10.1186/s13063-018-2939-2 (PMC6195975; doi:10.1186/s13063-018-2939-2)
Supplement: Supplementary file 2 — Structure and content of the four sessions. (PDF 1237 kb) [file 13063_2018_2939_MOESM2_ESM.pdf]

Movement through Active Personalised engagement MAP

Moving more

Key messages:
The benefits of moving more, both physical and psychological.
Barriers to moving more.
Strategies to enable moving more.
Personalising benefits to moving more and goal setting.

1 Thinking about my health 15mins

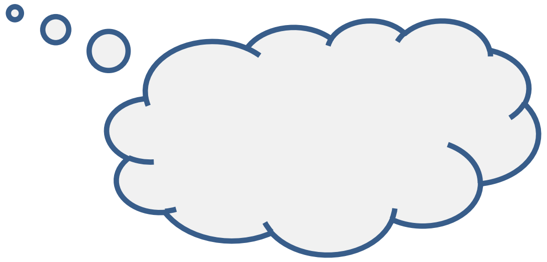

Collecting current thoughts:

- How does having multiple health conditions affect you?
- How could this programme make a difference to you?
- How could moving more or being more active help you?

2 The Group View 30mins

When they look at this/read this what are they thinking?

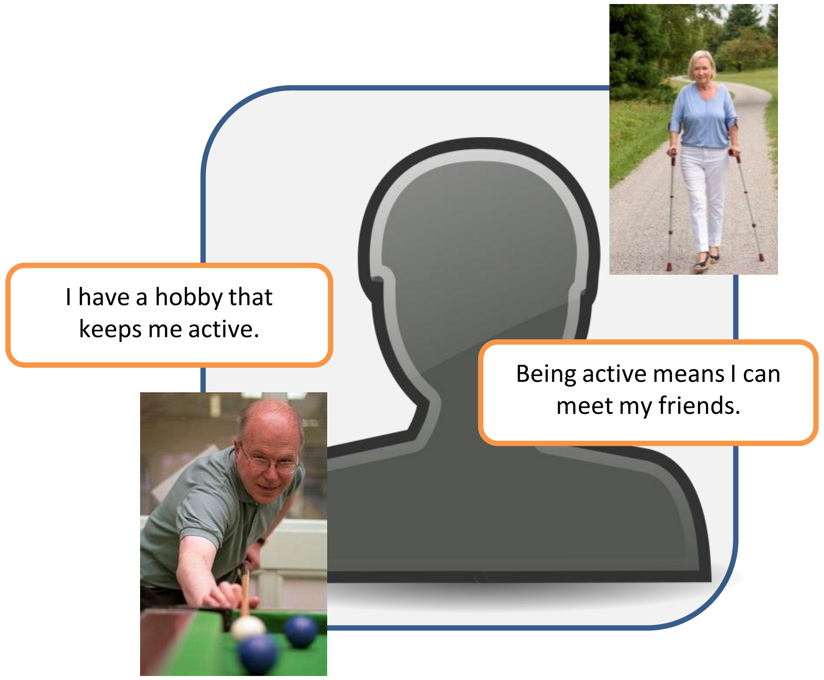

3 Personalising 35mins

my activity MAP

How could moving more be included into your day?

Stand up in advert breaks

I get up

go to bed

my Activity MAP

Activity Diary: What activity did I do this week?

Date started:

Day	Time	What did I do?	How long? or How many steps?
Monday			
Tuesday			

Activity Diary

my resource MAP

A space to record the places you can find further information or support.

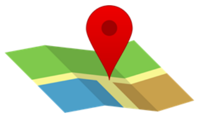

my resource MAP

Resources

Resource book

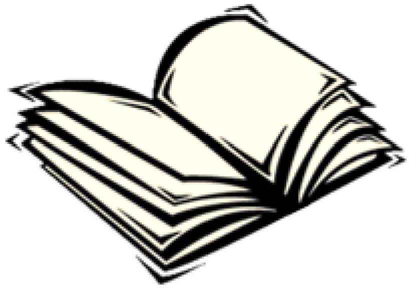

Using a pedometer and resistance band

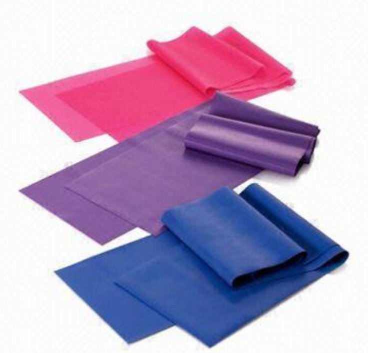

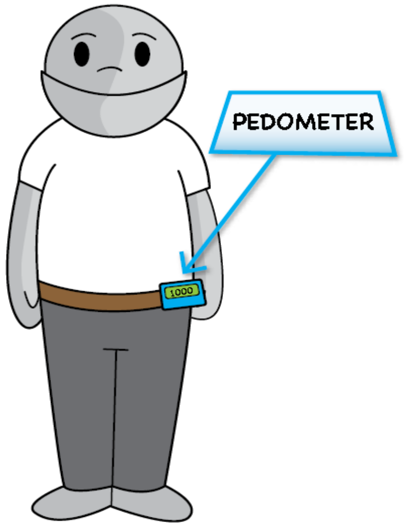

Movement through Active Personalised engagement MAP

Mastering emotions

1 Thinking about my health 15mins

Collecting current thoughts:

- What has changed?
- What are your thoughts about moving more now?

2 The Group View 30mins

When they look at this what are they thinking?

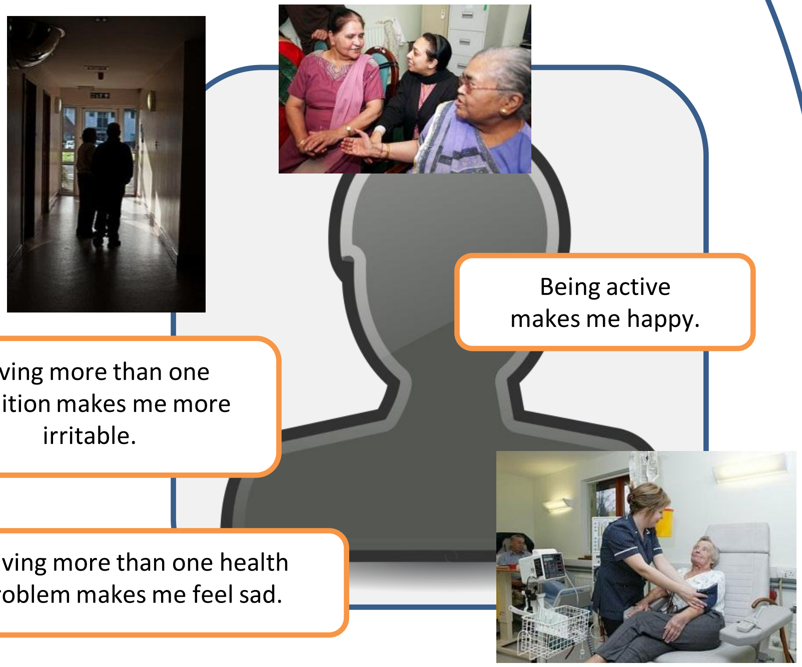

Key messages:

- Awareness of emotions and their impact on managing health.
- How health can affect emotions.
- Benefits of awareness of emotions, and how this can affect managing health.
- Personalising mastering emotions by identifying triggers and strategies.

Resources

Resource book

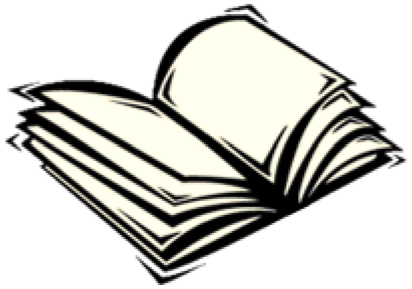

3 Personalising 35mins

Activity Diary: What activity did I do this week?

Date started:

Day	Time	What did I do?	How long? or How many steps?
Monday			
Tuesday			
Wednesday			
Thursday			
Friday			

Activity Diary

Review activity

my resource MAP

A space to record the places you can find further information or support.

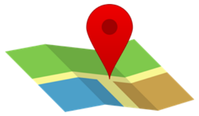

my resource MAP

Prompts to help people think about their feelings

- I have lost interest in things
- I get sort of frightened
- I look forward
- I can see the funny side of things
- I worry
- I feel tense or 'wound up'

Movement through Active Personalised engagement MAP

Managing treatments

Key messages:

- The benefits of managing treatments.
- Reasons for people not adhering to treatments.
- Confidence and importance of managing treatments.
- Strategies for managing treatments.

1 Thinking about my health 15mins

Collecting current thoughts:

- What has changed?
- What are your thoughts about moving more now?

2 The Group View 30mins

When they look at this what are they thinking?

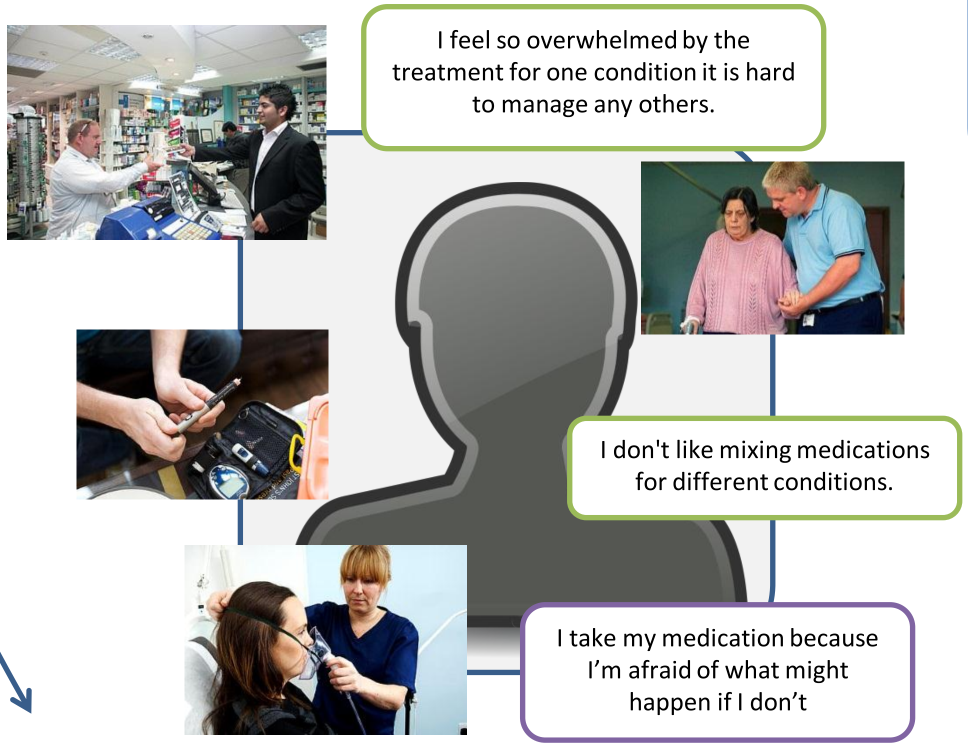

Resources

Resource book

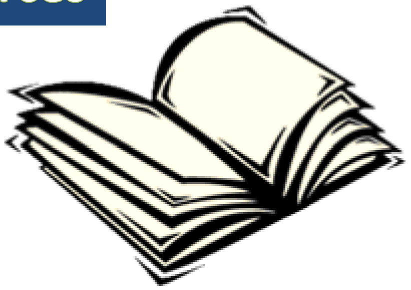

3 Personalising 35mins

Activity Diary: What activity did I do this week?			
Date started:			
Day	Time	What did I do?	How long? or How many steps?
Monday			
Tuesday			
Wednesday			
Thursday			
Friday			

Activity Diary

Review activity

How important is managing their treatments for their health?

How confident do they feel managing treatments?

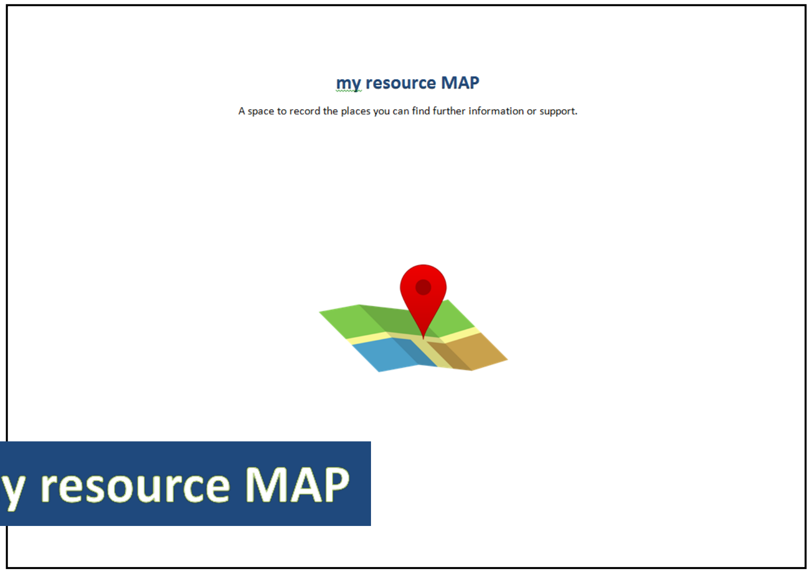
my resource MAP

A space to record the places you can find further information or support.

my resource MAP

Effective communication

Movement through Active Personalised engagement
MAP

1 Thinking about my health 15mins

Collecting current thoughts:

- What has changed?
- What are your thoughts about moving more now?

2 The Group View 30mins

When they look at this what are they thinking?

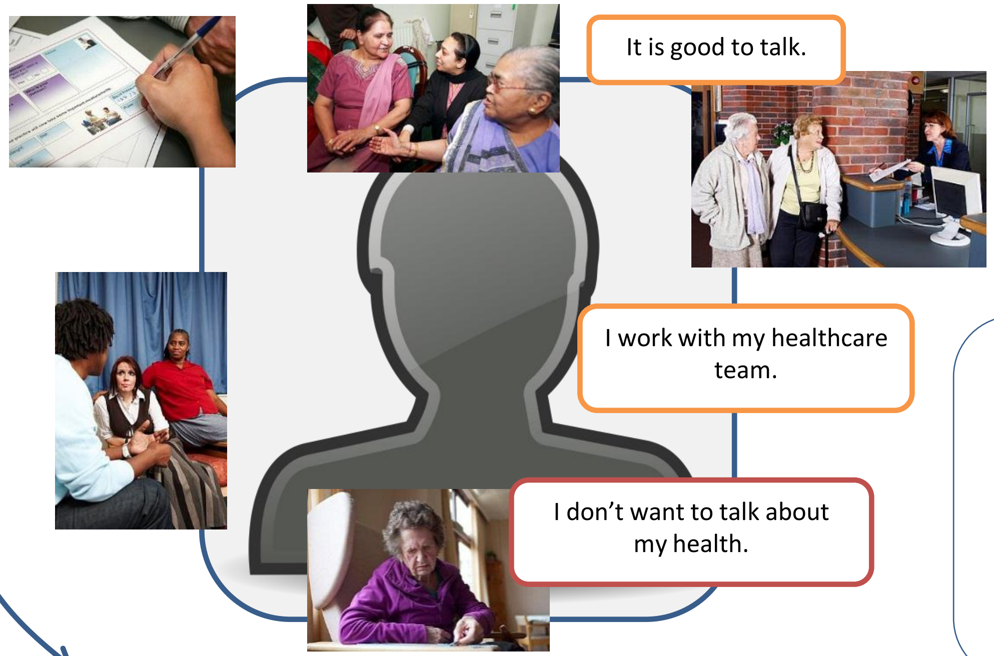

Key messages:

- Who is involved in your healthcare communication.
- Benefits of effective communication.
- Strategies for effective communication.
- Locating and using quality information.

Resources

Resource book

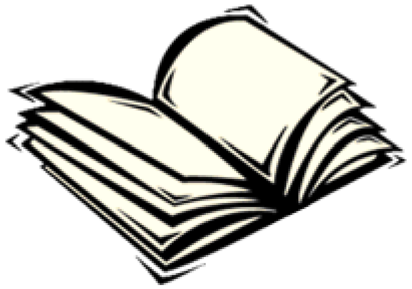

3 Personalising & Reviewing 35mins

Activity Diary: What activity did I do this week?

Date started:

Day	Time	What did I do?	How long? or How many steps?
Monday			
Tuesday			
Wednesday			
Thursday			
Friday			

Activity Diary

Review activity

Where could they go to find quality information?

Reviewing

What are **their** key messages from MAP?

my resource MAP

A space to record the places you can find further information or support.

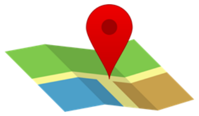

my resource MAP
