# Supplementary material for: Movement through Active Personalised engagement (MAP) — a self-management programme designed to promote physical activity in people with multimorbidity: study protocol for a randomised controlled trial
Source: Trials. 2018 Oct 20;19:576. doi: 10.1186/s13063-018-2939-2 (PMC6195975; doi:10.1186/s13063-018-2939-2)
Supplement: Supplementary file 3 — MAP follow-on support pathway. (PDF 590 kb) [file 13063_2018_2939_MOESM3_ESM.pdf]

MAP Follow-on Support Pathway

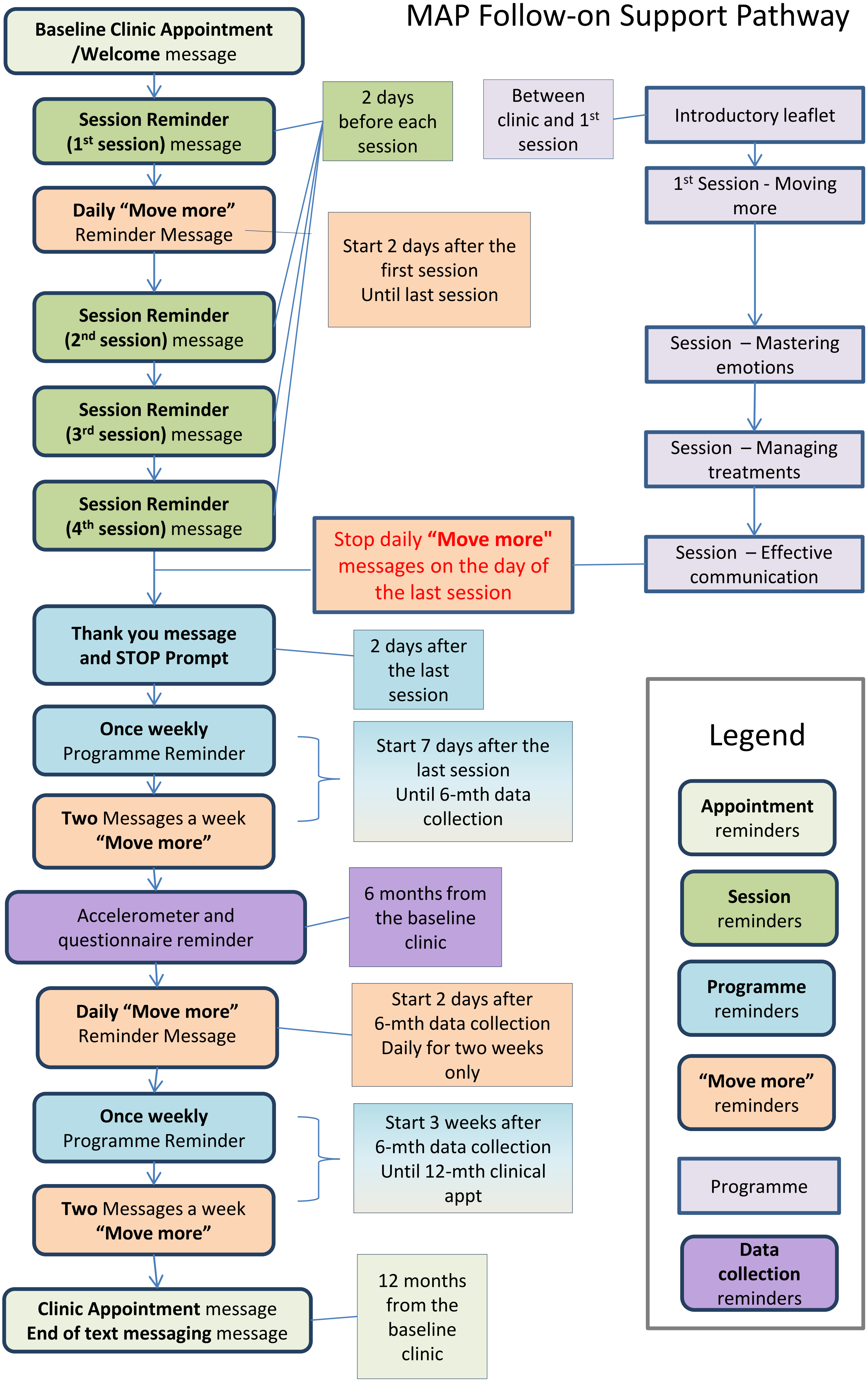

"move more" reminder messages, daily in random order (then repeat):

Hi…have you thought about moving more today?
Hi...don't forget every step counts.
Hi...how can you fit moving more into your day?
Hi...moving more can help prevent conditions from getting worse.
Hi...moving more can help you stay independent.
Hi...moving more can improve your sleep.
Hi...moving more can keep you mobile.
Hello...moving more can make a difference to you.
Hello...every step counts.
Hello...feel better by moving more.
Hello...moving more is one of the simplest ways to look after yourself.
Hello...keeping track of your steps?
Hello...moving more can help you feel positive.
Hello...try to reduce your sitting time.
Hello...when can you move more today?
Hello...why not set yourself a new goal to move more?
Hi...moving more can improve how you feel.
Hi...moving more can help your confidence.
Hi...why not try to reduce your sitting time?
Hi...why not try to break-up sitting time?
Hi...remember any activity is better than none.
Hi...moving more can promote better health.
Hi...why not keep track of your activity in a diary or activity MAP?

***Programme reminder message once weekly in this order (then repeat)** :*

Hi...have you used your resource MAP?
Hi... thinking about MAP...have you considered how thoughts or feelings impact on your life and health?
Hi... thinking about MAP...what would help you feel more confident about managing your health?
Hi... reflecting on MAP... what has helped you to manage your health?
Hi...thinking about MAP... have you reviewed your treatment goals recently?
Hi...have you MAPped your day? to see how treatments and emotions affect you?
Hi... thinking about MAP...when did you last review your treatment options?
Hi...don't forget you can speak to your doctor, pharmacist or healthcare team about health concerns.
Hi... reflecting on MAP...what can you do to get the best from your healthcare team?
Hi... thinking about MAP...why not take a look at NHS Choices www.nhs.uk
Hi...don't forget to use your resource MAP for quality information.
